# Triple Assessment of Breast Lump: Should We Perform Core Biopsy for Every Patient?

**DOI:** 10.7759/cureus.7479

**Published:** 2020-03-30

**Authors:** Muhammad Osman Karim, Kashuf A Khan, Abdul Jalil Khan, Ayesha Javed, Sheraz Fazid, Muhammad Imran Aslam

**Affiliations:** 1 General Surgery, Royal Shrewsbury and Telford Hospital National Health Service (NHS) Trust, Shrewsbury, GBR; 2 Family Medicine, Khyber Medical University, Peshawar, PAK; 3 General Practice, National Health Service (NHS), Bolton, GBR; 4 General Practice, Hospital of St Cross, Warwick, GBR; 5 Public Health, Khyber Medical University Hospital, Peshawar, PAK; 6 General Surgery, Warwick Hospital, Warwick, GBR

**Keywords:** triple assessment, breast cancer, core biopsy

## Abstract

Introduction

The triple assessment for a lump in the breast is standard practice and the robustness of assessment towards the diagnosis of breast cancer is crucial. The combination of the modalities, physical examination, imaging (mammogram and ultrasound), and fine-needle aspiration cytology (FNAC) is more accurate than any modality alone.

Aim

To examine the combined and individual predictive values of physical examination (P), mammography (M), ultrasound (U), FNAC (C), with core biopsy (B) - triple assessment in the diagnosis of breast cancer.

Methods

To obtain the results of physical examination (P), mammography (M), ultrasound (U), FNAC (C), and core biopsy (B), we examined the records of 124 breast cancer patients seen between April 1, 2009, and March 30, 2010. To assess the diagnostic potential of the combination of the modalities (P, U, and M), we considered all cases with a score of 4 (probably malignant) and 5 (malignant) as positive for malignancy. All cases with a score of 3 (equivocal), 2 (benign), and 1 (normal) were considered negative for malignancy. For FNAC, a score of 1 (insufficient sample), 2 (benign), and 3 (atypia/probably benign) were considered. All the patients were diagnosed with breast cancer on excision biopsy. Among 124 patients, 12 were excluded, as they were unfit for intervention.

Results

The accuracy of physical examination (P) as confirmed by core biopsy (B) is dependent on the experience of the surgeon. It has limitations in younger women and smaller lesions. In our study, P has a positive predictive value (PPV) of 58.9% when compared with surgical biopsy, which is comparable with other studies. Our results showed PPV 66.1% and after an ultrasound scan, the overall radiological grading (M & U) gives a PPV of 81.3%, reflecting the important role of ultrasound scans. Our results showed the sensitivity of FNAC to be 73.2%. Core biopsy was diagnostic in 107 (95.5%) patients, making it a reliable tool. Our results confirmed that a combination of the modalities (P, M, U, R, FNAC) is more accurate than any modality alone.

Conclusion

When all the three modalities are positive for a diagnosis of malignant breast disease, surgical biopsy confirms the breast cancer diagnosis with a PPV of 100% and a sensitivity of 95.5%.

## Introduction

The first written evidence of breast cancer dates back from 3000 to 2500 BC from ancient Egypt in the Edwin Smith Papyrus [1-2]. There is an increasing incidence of breast cancer; it was reported to have caused over a quarter (28%) of all the deaths in the UK in 2017 [3]. It is imperative to develop new approaches for the early detection of cancer to improve survival and to decrease the burden on health care professionals [4].

In the spectrum of symptoms related to breast disease, a breast lump is the most commonly presented symptom. It may either be a manifestation of benign pathologies, such as fat necrosis, fibroadenoma, acute or chronic breast abscess, or a sinister carcinoma breast [5].

Distinguishing between benign and malignant breast lesions solely by a clinical/physical examination is subjective and clinician dependent and carries a risk of uncertainty and error. Core biopsy is considered to be a reliable test used in the detection of breast cancer, however, it requires time and expertise and can be a painful experience. Most hospitals in the UK run “Rapid Access” breast cancer screening clinics offering triple assessment [6].

Triple assessment, as the name indicates, includes three modalities, physical examination, imaging (mammography and/or ultrasound), and biopsy (FNAC and core biopsy). These modalities, when used individually for breast cancer screening and diagnosis, will render less reliable results as compared to when used in combination.

Our study was done to assess the reliability, validity, sensitivity, and specificity of triple assessment and to compare it with core biopsy. The aim was to assess the accuracy of core biopsy (B) alone in diagnosing breast cancer as compared with the triple assessment.

## Materials and methods

This was a cross-sectional retrospective study conducted at Tameside General Hospital Manchester, UK, between April 1, 2009, and March 30, 2010. We examined the records of 124 breast cancer patients aged from 18 to 83 years, who were initially assessed in a One-stop Triple Assessment Clinic and core biopsy was performed either on the same day or the day after. Approval from the institution’s ethical committee was obtained and informed consent was taken from patients for physical examination and investigations.

The study was done to assess the reliability, validity, sensitivity, and specificity of triple assessment and to compare it with core biopsy. To assess the diagnostic potential of the combination modalities (P, U, and M), we considered all cases with a score of 4 (probably malignant) and 5 (malignant) in any of the modalities positive for malignancy. All cases with a score of 3 (equivocal), 2 (benign), and 1 (normal) in any of the modalities were considered negative for malignancy. For FNAC, a score of 1 (insufficient sample), 2 (benign), and 3 (atypia/probably benign) were considered. All patients included in the study were diagnosed to have breast cancer on surgical biopsy. Among 124 patients, 12 were excluded. The reason for exclusion was unfitness for intervention, which is explained below in detail:

Nine patients were too frail and unfit who did not undergo the full triple assessment:

One patient was started on letrozole without triple assessment (patient’s wish, 83-year-old).

One patient had ulcerated and fungating breast cancer of the nipple-areola complex.

One patient failed to attend the clinic for a surgical biopsy despite reminders.

Each patient went through a physical examination of breast bilaterally. Skin changes, discharge or bleeding from the nipple, and lumps were noticed during the examination. All characteristics of a lump were recorded such as location, size, shape, edges, mobility, adherence to the skin or underlying structures and tenderness. Along with breast examination, axilla and supraclavicular fossae were examined bilaterally for assessment of lymphadenopathy, and any signs of distant metastasis were examined.

Mammography was performed according to standard guidelines, a craniocaudal view of each breast was taken, and two views were obtained: the lateral oblique view and a view with the tube angled at 45 degrees to the horizontal axis. To obtain these views successfully during mammography, the nipple should be seen in profile, the anterior surface of the pectoralis major should be visible, the breast should be lifted sufficiently and is compressed between compression plate and film to have evenly spread breast tissue, as it makes it easy to detect any changes. Irregular borders, micro-calcifications, speculated density, loss of breast architecture, and skin retraction suggests malignant disorder while a well-circumscribed mass with regular borders is suggestive of benign disorders.

High-definition ultrasonography breast (HDUSG) was used during this study. The patient should lie in a supine or oblique position, with the ipsilateral arm above the head. The breast has to be scanned in either a transverse or sagittal or radial and anti-radial planes. The transducer is used in multiple planes to evaluate the retro-areolar area, as it is shadowed by nipple artifact on ultrasound.

FNAC was done in all patients using a 22 gauge needle and 20 ml syringe. The mass was immobilized and the needle was inserted into the breast lump. The needle was moved back and forth inside the mass and the material was then expelled onto a glass slide, fixed by air drying, and stained with Giemsa and hematoxylin and eosin. Slides taken from patients were examined by the pathologist and the cytological diagnosis of the breast masses was given.

## Results

After statistical analysis by comparing each modality with the core biopsy and then collectively compared with the core biopsy, the following results were obtained as shown in the tables below.

Clinical examination versus core biopsy

On physical examination, 45 (40.2%) patients were assessed negative and 65 (58.0%) of patients had breast cancer. There were two false-positive results and no false-negative result (Table 1).

**Table 1:** Clinical Examination vs. Core Biopsy

CLINICAL EXAMINATION	CORE BIOPSY		
	Positive	Negative	Total
Positive	65	2	67
Negative	0	45	45
Total	65	47	

And in the end, when compared to the core biopsy, we concluded that physical examination had 97% sensitivity, 96% specificity, 97% positive predictive value, and 100% negative predictive value.

Radiological assessment versus core biopsy

After mammography, 73 (65.2%) patients were considered to have breast cancer and after an ultrasound scan, the combined radiological grading (M and/or U) was able to diagnose 90 (80.3%) patients as having carcinoma of the breast. Twenty (0.18%) patients tested to be true negative; one of the patients had a false-negative result and one had a false-positive result, which indicated that the sensitivity of radiological assessment is tested to be 99%, specificity 95.2%, positive predictive value 99% and negative predictive value 95% (Table 2). All the patients had undergone FNAC thereafter.

**Table 2:** Combined Radiological Assessment vs. Core Biopsy

COMBINED RADIOLOGICAL ASSESSMENT	CORE BIOPSY		Total
	Positive	Negative	
Positive	90	1	91
Negative	1	20	21
Total	91	21	

FNAC versus core biopsy

FNAC was successful in diagnosing 82 (73.2%) patients with breast cancer; 29 (25.9%) patients were tested negative for cancer while one false-negative result and no false-positive results were obtained. This reflects FNAC has sensitivity, specificity, a positive predictive value, and a negative predictive value of 98.8%, 100%, 100%, and 97%, respectively.

There were 49 (43.7%) patients who were found positive in all three modalities (P, M, and C) of the triple assessment.

Core biopsy results

Among all of the patients, core biopsy was able to diagnose 107 (95.5%) patients with breast cancer (Table 3). Cases that were equivocal or benign on core biopsy were positive on triple assessment. (P, M, and C).

**Table 3:** Core Biopsy

BIOPSY RESULTS	NO. OF PATIENTS
MALIGNANT CARCINOMA	107
BENIGN TUMOR	3
EQUIVOCAL	2
TOTAL PATIENTS	112

Triple assessment versus core biopsy

The combination of all the modalities gave a positive predictive value of 100% when compared with a surgical pathological diagnosis (Table 4).

**Table 4:** Triple Assessment vs. Core Biopsy

TRIPLE ASSESSMENT	CORE BIOPSY		
	Positive	Negative	Total
Positive	90	0	90
Negative	5	17	22
Total	95	17	

## Discussion

Triple assessment is considered positive when any one of the three modalities among clinical examination of breast, imaging, and histology is positive and is deemed negative when all three modalities are negative.

Our results confirmed that a combination of all modalities (P, M, U, and FNAC) is more accurate than individual modality alone. When all the three modalities were positive for a diagnosis of malignant disease, the surgical biopsy also confirmed the diagnosis. In our study, physical examination (P) detected breast carcinoma in 65 patients, 90 patients were tested positive in the radiological assessment, and 82 patients had positive FNAC while 107 patients had malignant changes in core biopsy. In a nutshell, the triple assessment had a positive predictive value of 100%, a negative predictive value of 77%, sensitivity of 94.7%, and specificity of 100%, which is comparable to other large studies as given below.

A study conducted by Lod Khoda et al. reveals the sensitivity, specificity, positive predictive value, negative predictive value, and accuracy of the physical examination were 66.6%, 100%,100%, 90%, and 91.6%, respectively. Various other studies also show the sensitivity of physical examination ranging from 21% to as high as 100% and the specificity from 50% to 97.8% [7]. Pande et al. in 2003 found the sensitivity, specificity, positive predictive value, and negative predictive value for ultrasound was 95%, 94.10%, 95.50%, and 93.75%, respectively [8]. In another study by Jan et al., ultrasound of breast lumps showed sensitivity, specificity, positive predictive value, and negative predictive value of 100%, 96.4%, 66.7%, and 100%, respectively [9]. Yang et al. (1996) found that sensitivity, specificity, and positive predictive value for ultrasound was 97% and 85%, respectively [10].

The sensitivity, specificity, positive predictive value, negative predictive value, and accuracy of FNAC were 91.6%, 100%, 100%, 97.4%, and 98%, respectively. Muhammed et al. in their study found the sensitivity, specificity, positive predictive value, and negative predictive value of FNAC were 90.6%, 100%, 100%, and 99% respectively [11]. Khemka et al. found that FNAC had the sensitivity, specificity, positive predictive value, and negative predictive value of 96%, 100%, 100%, and 95.12%, respectively [12]. Core biopsy sensitivity, specificity, positive predictive value, and negative predictive value of 94.7%, 100%, 100%, and 77%, respectively.

Our results are comparable to other major studies like Ghafouri et al., who also found that when suspicious results were excluded, the diagnostic accuracy of the modified triple assessment was 100% [13]. When the triple assessment was compared with the results of histopathology another study states that the concordance for the triple test was 99.3%, specificity was 100%, and sensitivity was 99.3%. Positive predictive value was 93.3%, negative predictive value was 100%, and p-value was significant (0.000) [14]. In another study by Janet et al., the sensitivity and specificity of triple assessment were 100% and 99.3%, respectively. The concordance for the triple assessment was 99.3%, positive predictive value was 93.3%, and the negative predictive value was 100% [15]. Similar findings were noted by Vaithianathan et al. in their study, the diagnostic accuracy rate of the modified triple assessment in concordant cases (benign and malignant) was 100%. The sensitivity, specificity, positive predictive value, and negative predictive value were found to be 100%, 82%, 76.9%, and 100%, respectively [16].

Our study suggests that although breast cancer can be confirmed without core biopsy, core biopsy gives additional information in treatment planning if the results confirm malignancy. FNAC is useful for the diagnosis as well as detection of estrogen and progesterone receptor status, however, human epidermal growth factor receptor 2 (HER-2) status cannot be determined by FNAC alone.

## Conclusions

Our study concludes that when all the three modalities (P, M and/or U, and FNAC) are positive for a diagnosis of malignant breast disease, core biopsy confirms the breast cancer diagnosis with a PPV of 100% and a sensitivity of 94.7%. In other words, if all three modalities give negative results, breast cancer is excluded and the patient can be safely discharged. We suggest that instead of putting the patient through pain and agony of biopsy, the three modalities of triple assessment (P, M and/or U, and FNAC) can be safely done to assess the status of cancer, and it can be a potential alternative to core biopsy.
